# Single Cell RNA-Sequencing Reveals a Murine Gallbladder Cell Transcriptome Atlas During the Process of Cholesterol Gallstone Formation

**DOI:** 10.3389/fcell.2021.714271

**Published:** 2021-09-28

**Authors:** Jingjia Liang, Wentao Shao, Qian Liu, Qifan Lu, Aihua Gu, Zhaoyan Jiang

**Affiliations:** ^1^Center of Gallbladder Disease, Shanghai East Hospital, Institution of Gallstone Disease, School of Medicine, Tongji University, Shanghai, China; ^2^Key Laboratory of Modern Toxicology of Ministry of Education, State Key Laboratory of Reproductive Medicine, Center for Global Health, School of Public Health, Nanjing Medical University, Nanjing, China; ^3^School of Instrument Science and Engineering, Southeast University, Nanjing, China

**Keywords:** cholesterol gallstone, gallbladder, single-cell RNA sequencing, lithogenesis, ursodeoxycholic acid

## Abstract

Gallstone disease is a worldwide common disease. However, the knowledge concerning the gallbladder in the pathogenesis of cholesterol gallstone formation remains limited. In this study, using single-cell RNA sequencing (scRNA-seq) to obtain the transcriptome of gallbladder cells, we showed cellular heterogeneity and transcriptomic dynamics in murine gallbladder cells during the process of lithogenesis. Our results indicated gallbladder walls were subjected to remodeling during the process of lithogenesis. The major molecular events that happened included proliferation of epithelial cells, infiltration of immune-cells, activation of angiogenesis, and extracellular matrix modulation. Furthermore, we observed partial reversal of gallbladder cell transcriptomes by ursodeoxycholic acid treatment. This work thus provides novel and integral knowledges on the cellular changes during lithogenesis, which is of great significance to the understanding of pathogenesis and treatment of cholesterol gallstone.

## Introduction

Gallstone disease is a worldwide common disease especially in Western countries (Cen et al., 2018; Kang et al., 2018). More than 80% of gallstones are of cholesterol type. Gallbladder is the site where gallstone forms, and its role in promoting gallstone formation has been investigated for decades (Bouchier, 1992). Many studies focused on the involvement of epithelial wall of gallbladder in the pathogenesis of gallstone, e.g., water absorption through aquaporin proteins (Paumgartner and Sauerbruch, 1991), epithelial transportation of cholesterol (via Abca1, g1, g5/g8) (Di Ciaula et al., 2018), and mucin secretion and modification of the balance of pro-/anti-nucleation factors (Bar Dayan et al., 2004). Defects in smooth muscle cell function has long been recognized as an important factor involved in promoting gallstone formation as well (Portincasa et al., 2000). However, the human gallbladders obtained during the operation were usually at the stage when gallstones were already formed, which more represented a later stage of changes in gallbladder wall or the resulting status when epithelial wall and gallstones are interacting for a long time. Furthermore, due to the wide application of laparoscopic cholecystectomy as a golden standard in treating gallstone disease, the role of gallbladder has been somewhat neglected. The knowledge concerning the gallbladder in the pathogenesis of gallstone formation still remains limited.

Single-cell RNA sequencing (scRNA-seq) is a recent rapid developed technique enabling the use of single cell transcriptional profiles to explore cellular events during the process of organ development (Chen et al., 2018; Bautista et al., 2021; Wu et al., 2021) as well as disease progress (Goveia et al., 2020; Rohlenova et al., 2020; Tombor et al., 2021). This technique allows the understanding of cellular heterogeneity and dynamic changes within an organ and during the development of certain disease (Chung et al., 2017; Tombor et al., 2021).

In this study, using sc-RNA sequencing of gallbladder cell samples, we reported the cellular heterogeneity and transcriptomic dynamics in murine gallbladder cells, providing a novel and integral understanding of the cellular changes during lithogenesis. We also identified major events in epithelial and non-epithelial cells during the process of lithogenesis and in response to ursodeoxycholic acid treatment. These events included proliferation of epithelial cells, infiltration of immune-cells, activation of angiogenesis, and extracellular matrix modulation.

## Materials and Methods

### Animal Experiment Process

Adult male C57BL/6J mice (8 weeks of age, weighted at 20 ± g) were purchased from Shanghai SLAC Laboratory Animal Co., Ltd. Shanghai, China. After acclimating to the environment, mice were randomly allocated into groups fed with lithogenic diet (LD: containing 1% cholesterol and 0.5% cholic acid) (Chen et al., 2019) or LD+0.5% tauroursodeoxychlic acid (TUDCA) (Lu et al., 2020). Diet and water were obtained *ad libitum*. All mice were housed at 24°C ± 2°C with a 12-h light-dark cycle in the animal facility. Mice were sacrificed at day 0, day 10, and day 28 of LD and day 28 of LD + TUDCA, respectively (*n* = 15 mice/time point). Before sacrifice, mice were fasted overnight and were sacrificed between 9 and 10 a.m. Cystic duct was clamped and gallbladder was removed from each mouse and processed for the isolation of gallbladder cells. All the study protocol was approved by the Ethical Committee at Shanghai East Hospital.

### Isolation of Cells From Mouse Gallbladder

When sacrificed, mice were anesthetized using phenobarbital. Upon opening the abdomen, the gallbladder was exposed and cystic duct was ligated. The gallbladder was soon removed avoiding liver tissues at the gallbladder bed. After aspiration of the bile, the gallbladder was washed with D-Hanks solution. Thereafter, the gallbladder was cut into pieces and put in 1.5 ml Trypsin-EDTA (0.25%) solution (No. 25200-056, Gibco^®^) and incubated at 37°C for 20 min and then centrifuged at 1500 rpm for 5 min. After removing the supernatant, the pellet was suspended in Trypsin-EDTA solution and repeated the digestion process two more times. The collected cell pellets were then suspended in Gibco^®^ RPMI 1640 medium. The living singles cells were then picked by a mouth pipette and transferred to cold scRNA-seq lysis buffer (Vieira Braga and Miragaia, 2019; Denisenko et al., 2020). To minimize experimental variation, all the procedures were performed by one expertise researcher.

### Single Cell RNA Sequencing and Data Analysis

#### Cell Preparation, Gel Beads in Emulsion Creation, and cDNA Amplification

The single-cell RNA-sequencing and data analysis processes were fulfilled at Genergy Biological Technology Limited Co. (Shanghai, China) according to standard protocols. For the quality check and counting of single cell suspension, the cell survival rate was more than 80%. The cells that had passed the test were washed and re-suspended to prepare a suitable cell concentration of ∼1,000 cells/μl for 10x Genomics ChromiumTM. The loaded Single Cell 3′ Chip was placed on a 10X Genomics Chromium Controller Instrument (10X Genomics, Pleasanton, CA, United States) to generate single cell gel beads in emulsion (GEMs). GEMs were constructed for single cell separation according to the number of cells to be harvested. After GEMs were normally formed, GEMs were collected for reverse transcription in a PCR machine for labeling. The GEMs were oil-treated, and the amplified cDNA was purified by magnetic beads and then subjected to cDNA amplification and quality inspection.

#### Library Preparation, Quantification, and Sequencing

The 3′ Gene Expression Library was constructed with the quality-qualified cDNA. After fragmentation, adaptor ligation, sample index PCR, etc., the library is finally quantitatively examined. Single cell RNA-seq libraries were prepared using the Chromium Single Cell 3′ Library & Cell Bead Kit (Cat. Nos. 120237, 120236, 120262; 10X Genomics) according to the manufacturer’s protocol. The final library pool was sequenced on the Illumina instrument using 150-base-pair paired-end reads.

#### Differentially Expressed Genes From Single-Cell RNA-Sequencing

The Cell Ranger Single Cell Software Suite was used to perform sample de-multiplexing, alignment, filtering, and UMI counting^1^. An algorithm based on mutual nearest neighbors (MNN, Haghverdi et al., 2018) was used to correct the batch effects. Clustering and gene expression were visualized with 10X Genomics Loupe ^R^ Cell Browser v.2.0.0^2^. The mean and variance of gene expression density, cluster identities, and filtered gene matrices generated by Cell Ranger^3^ were used as input into R toolkit Seurat^4^ (Satija et al., 2015) for generating violin plots. Cell–cell interaction analysis was performed by CellPhone method according to the standard protocol (Efremova et al., 2020).

#### Gene Set Variation Analysis

In the GSVA, gene sets were obtained from the ‘HALLMARKS’ collection of the MSigDB database, publicly available at http://www.broadinstitute.org/gsea/msigdb. In the analysis, the signature enrichment scores of individual single cells were calculated (Hanzelmann et al., 2013). The resulted GSVA score matrix was organized as signatures in the rows and single cells in the column. Comparisons of single cell enrichment scores were performed using R package ‘GSVA.’ Differentially enriched signatures were defined as having FDR adjusted *p*-values < 0.05 and mean score difference ≥ 0.1 as described (Gao et al., 2017; Kim et al., 2018).

### Data and Software Availability

The raw data files were deposited at GEO repository (access No. GSE17924^5^).

The software and scripts used in this study were publicly available at the following websites: The Chromium Single Cell Software Suite: https://support.10xgenomics.com/single-cell-gene-expression/software/overview/welcome; running cell ranger agrr: https://support.10xgenomics.com/single-cell-gene-expression/software/pipelines/latest/using/aggregate; Seurat package: http://satijalab.org/seurat/install.html; pheatmap: https://cran.r-project.org/web/packages/pheatmap/index.html; topGO: http://www.bioconductor.org/packages/release/bioc/html/topGO.html; igraph: https://cran.r-project.org/web/packages/igraph/index.html; GSVA analysis: https://www.bioconductor.org/packages/release/bioc/html/GSVA.html, and computational code for CellPhone interaction analysis: https://github.com/Teichlab/cellphonedb.

## Results

### Single Cell Transcriptome Analysis of Gallbladder Identified Different Cell Types

**FIGURE 1:**
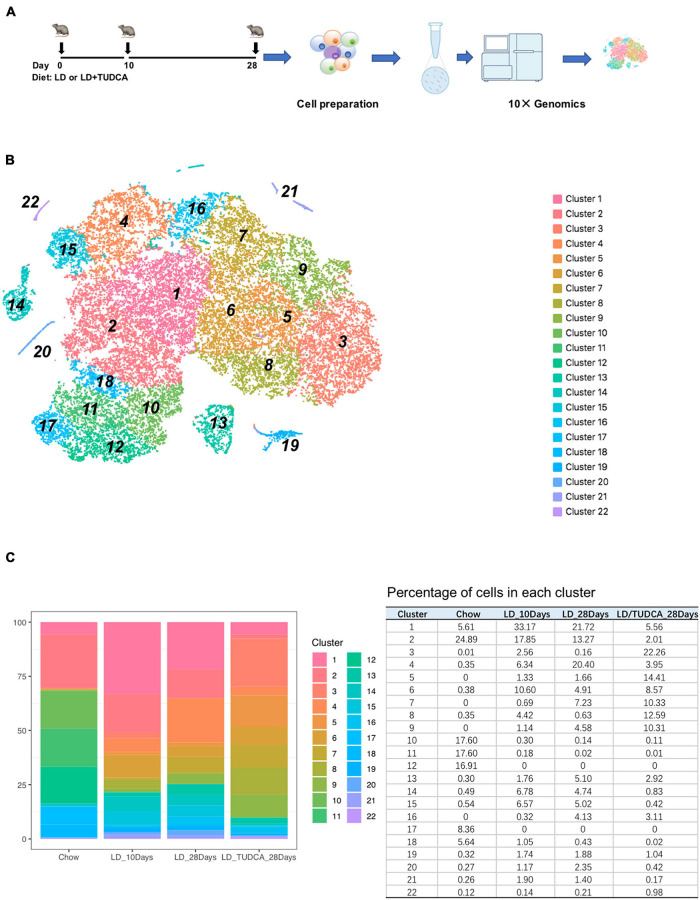
The transcriptomic heterogeneity of murine gallbladder. **(A)** The experimental design of mouse exposure to lithogenic diet (LD) or LD+ tauroursodeoxychlic acid (TUD). Mice were sacrificed at day 0 (chow diet, basal condition), day 10, and day 28 of LD and day 28 of LD + TUD, respectively (*n* = 15 mice/time point). At each time point, gallbladders were harvested and cell suspension were prepared and processed for 10 × genomic single cell sequencing (scRNA) under the standard protocols. **(B)** tSNE plot identified 22 clusters of gallbladder cells from scRNA sequencing data. **(C)** Stacked bar graph (left) and table (right) showing proportions of gallbladder cells in each cluster at each time point during process of cholesterol gallstone formation.

To understand the changes of cellular transcriptome of gallbladder cells during the process of cholesterol formation, the cells of gallbladder were harvested at day 0 (chow diet), day 10, and day 28 after fed with lithogenic diet (LD, Figure 1A). Day 0 represented the basal condition of gallbladder under chow diet, day 10 represented the acute phase after LD feeding, and day 28 represented the chronic phase after LD feeding when gallstones formed (Gilat et al., 2001). Tauroursodeoxycholic acid, as a modulator of bile acid hydrophobicity, has the capacity to partly reduce the incidence of gallstone formation in mice (Lu et al., 2020). Therefore, gallbladder cells from mice with day 28 LD + TUDCA feeding were also collected for the analysis to evaluate the effect of TUDCA on gallbladder.

After filtration (Xu et al., 2021) and exclusion of red blood cells and contaminated hepatocytes, a total of 35,654 individual cells were obtained for the subsequent analysis (Supplementary Figure 1). On average, each cell expressed 2,464 genes (median number) and 8,150 median UMI counts.

**FIGURE 2:**
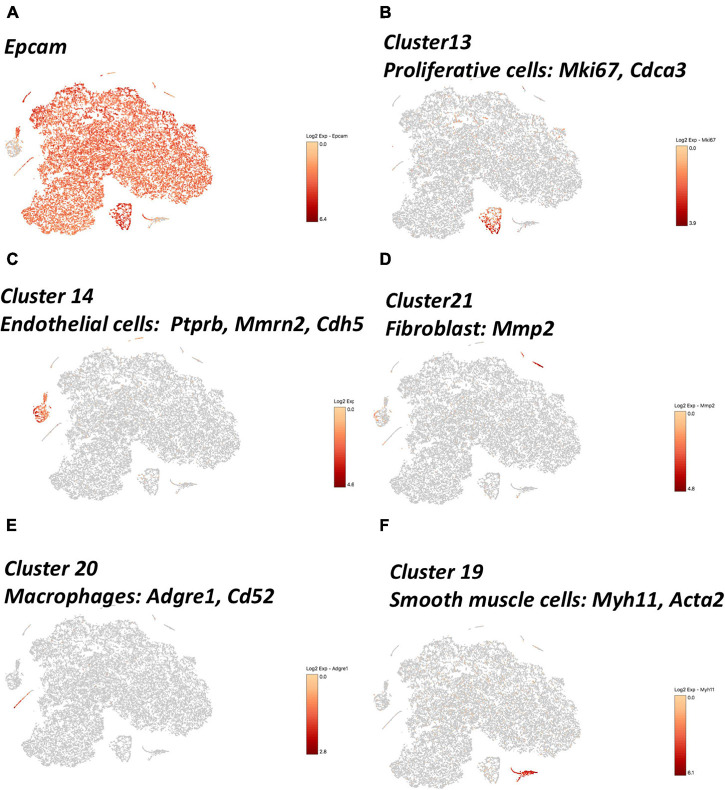
t-SNE plot showing the expression of known marker genes indicating cell types. **(A)** epithelial cells, *Epcam*; **(B)** proliferating cells, *Mki67*, *Cdca3*; **(C)** endothelial cells (ECs), *Ptprb*, *Mmrn2*, *Cdh5*; **(D)** fibroblast (FBs), *Mmp2*; **(E)** macrophages, *Adgre1*, *Cd52*; **(F)** smooth muscle cells, *Myh11*, *Acta2.*

Using the 10× genomics software, the aggregated and normalized data were analyzed to identify cell types by graph-based or k-means clustering and the 22 identified clusters were visualized by t-distributed stochastic neighbor embedding (t-SNE) plots (Figure 1B and heatmap show in Supplementary Figure 2). The composition of cells in each cluster was shown in Figure 1C. The major cell populations were epithelial cells of the gallbladder which were *Epcam* positive (Manohar et al., 2011; Figure 2A) and which were comprised of 98.5, 88.3, 89.1, and 96.6% at day 0, day 10, day 28, and day 28 + TUDCA, respectively. These cells were also enriched in makers for mucin secretion (Lee and Liu, 2002) (*Muc3*, *Muc4*) and water channel (Laforenza, 2012) (*Aqp1*), etc. (Supplementary Figure 3). Among the epithelial cells, a fraction of cells, cluster 13, belonged to highly proliferating cells according to cell makers such as *Mki67* (Sobecki et al., 2016), *Cdca3* (Kim and Bahk, 2014), *Cdc20* (Kim and Yu, 2011), and *Ccnb2* (De Martino et al., 2018; Figure 2B and Supplementary Figure 4A). Accordingly, they were enriched of GO terms as cell division (GO:0051301, *p* = 3.90E–14), cell cycle (GO:0007049, *p* = 4.00E–11), mitotic cell cycle (GO:0000278, *p* = 1.40E–08), indicating them to be proliferative cells (Supplementary Table 1). The clustering of cells by their transcriptomes also indicated high heterogeneity of the epithelial cells in gallbladder during different stages after diet feeding.

Four distinctive non-epithelial cell population were identified using canonical markers (Figures 2C–F and Supplementary Figures 4B–F). Cluster 14 was identified as endothelial cells [ECs, markers: *Ptprb, Mmrn2, Cdh5* (Der et al., 2017)], Cluster 21 as fibroblast cells [FBs, *Mmp2* (Rodriguez et al., 2010)], Cluster 20 as macrophages [*Adgre1, Cd52* (Zhao et al., 2020)], Cluster 19 as smooth muscle cells [*Myh11, Acta2* (Liu and Gomez, 2019)]. Gene ontology (GO) enrichment analysis of gene expression in each cluster further supported their predicted identities (Supplementary Table 1). For example, in endothelial cells, GO terms of vasculature development (GO:0001944, *p* = 1.2E–19), blood vessel development (GO:0001568, *p* = 2.2E–19), and angiogenesis (GO:0001525, *p* = 7.4E–19) were enriched. Fibroblast cells were enriched of terms associated with regulation of locomotion (GO:0040012, *p* = 9.7E–21), extracellular matrix organization (GO:0030198, *p* = 3.5E–19), extracellular structure organization (GO:0043062, *p* = 3.9E–19), and regulation of cell mobility (GO:2000145, *p* = 3.3E–18). Macrophages were associated with immune response (GO:0006955, *p* = 1.1E–27), immune system process (GO:0002376, *p* = 1.9E–26), and defense response (GO:0006952, *p* = 1.70E–23), and smooth muscle cells with actin filament-based process (GO:0030029, 1.10E–11), muscle contraction (GO: GO:0006936, *p* = 5.40E–10), and muscle tissue development (GO:0060537, *p* = 5.40E–09).

### Single-Cell RNA Sequencing Revealed Dynamic State of Gallbladder Cells During the Process of Lithogenesis

**FIGURE 3:**
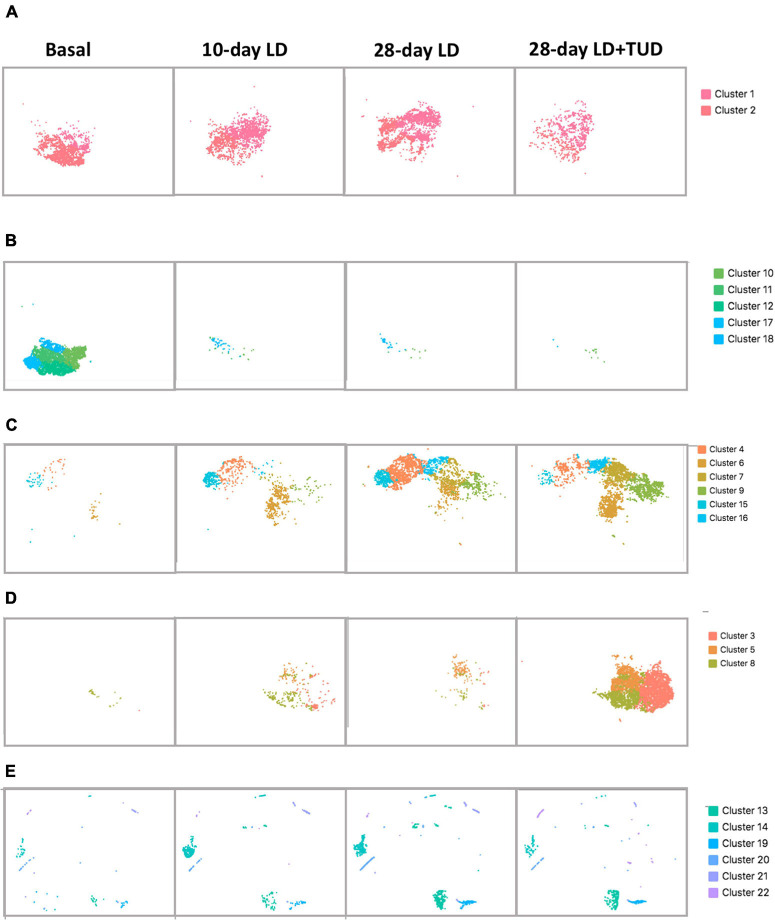
Shifting of gallbladder cell clusters during lithegenesis. t-SNE plot demonstrating the dynamic transcriptomic patterns during the process of lithogenesis and with TUDCA treatment. Basal (day 0), day 10, and day 28 gallbladder cells during gallstone formation. **(A–D)** Epithelial cells. **(E)** Non-epithelial cells. **(A)** Clusters 1 and 2 were shared by all diet conditions suggesting the constant preserved clusters of epithelium cell in gallbladder irrespective of diet challenge. **(B)** Clusters 10, 11, 12, 17, and 18 were almost exclusively present under basal condition and disappeared after LD. **(C)** Clusters 4, 6, 7, 9, 15, and 16 appeared in response to LD. **(D)** Clusters 3, 5, and 8 were more related with TUDCA treatment. **(E)** Clusters 13, 14, 19, 20, 21, and 22 were non-epithelial cells changed under lithogenic diet and with TUDCA treatment.

The overall cell analyzed by t-SNE plot also revealed the dynamic transcriptomic patterns during the process of lithogenesis and under TUDCA treatment. Cluster 1 and 2 were shared by all diet conditions representing the constant preserved clusters of epithelium cell in gallbladder irrespective of diet challenge (Figure 3A). The rest of the epithelial cells were apparently sensitive to diet which shifted away from basal condition indicating changes in response to LD feeding. Cluster 10, 11, 12, 17, and 18 were almost exclusively present under basal condition (Figure 3B) and disappeared after LD. Cluster 4, 6, 7, 9, 15, and 16 appeared with LD challenge (Figure 3C). Cluster 3, 5, and 8 were more related with TUDCA treatment (Figure 3D).

At basal condition, the gallbladder wall was usually lined with almost a single layer of cells and flat with few protrusions. This was in line with very few proliferative epithelial cells indicating the less active but more mature of cells under basal condition (cells in cluster 13, Figure 3E), suggesting that at basal condition, most epithelial cells were mature, static, and less differentiative. Lithogenic diet progressively induced proliferation of epithelial cells (cluster 13, Figure 3E), the composition of which increased from 0.3 to 1.76% at day 10 and climbed up to 5.1% at day 28, but reduced to 2.92% at day 28 under TUDCA treatment (Figure 1C). These data indicated that epithelial cell proliferation can be one of the major events for gallbladder during the process of lithogenesis.

#### Single-Cell RNA Sequencing Transcriptomes Suggested Damage and Repairing of Epithelial Cells

Cluster 4, 15, and 16 were progressively increased with time of LD (Figure 3C). GO enrichment analysis (Supplementary Table 1) indicated the expression of genes related with cellular response to chemical stimulus, response to oxygen-containing compound, response to lipids and regulation to cell death in cluster 4, regulation of cell proliferation, response to lipid, response to oxygen-containing compound in cluster 15, and protein refolding, response to stress, response to toxic substance, response to lipid in cluster 16. These clusters of epithelium cells indicating the cellular changes in response to changes of biliary chemical composition after LD with time. In contrast, TUDCA supplementary partly reversed this effect due to its modification of bile hydrophobicity and lipid composition.

Furthermore, cluster 3, 5, 8 were cells at status more related with TUDCA, though small numbers of these clusters were present after LD (Figure 3D). The GO enrichment analysis (Supplementary Table 1) indicated the presence of apoptic process, programmed cell death, and wound healing process in all these clusters. These findings of epithelial cells provided evidences that injury of gallbladder mucosa occurred before stones developed as proposed (Scott, 1978). In contrast, TUDCA could exert a role in the repairing process.

#### Gene Set Variant Analysis of Epithelial Cell Sub-Clusters

**FIGURE 4:**
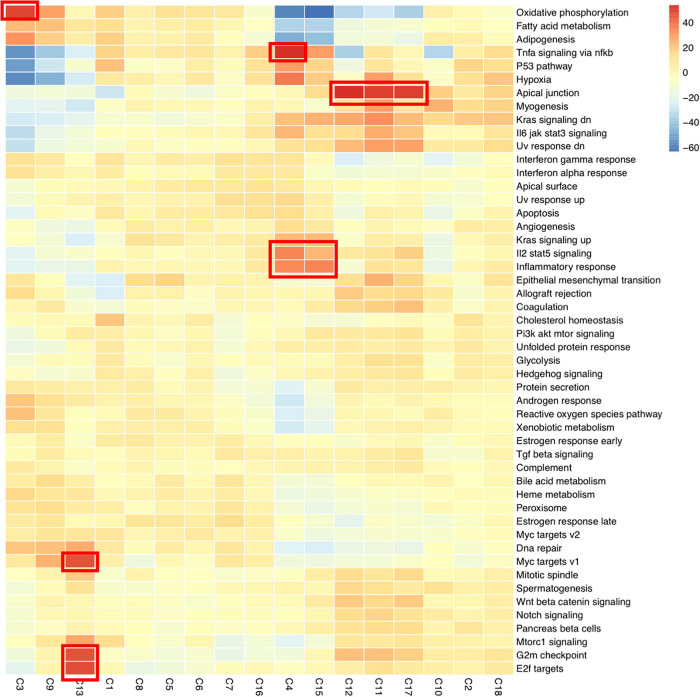
The heatmap showing the average expression of known marker genes related in in each epithelial cluster (cells under chow diet) by Gene Set Variant Analysis (GSVA). Clusters 11, 12, and 17 (cells under chow diet) were enriched in genes related with apical junction. Cluster 13 was enriched in genes of G2m checkpoint, E2f targets, Myc targets V1. Clusters 4 and 15 were enriched in genes involved in inflammatory response, Il2 stat5 signaling, Tnfα singling via Nfκb. Cluster 3 was enriched in oxidative phosphorylation.

To gain further understanding of the function of epithelium clusters, we performed GSVA. Clusters 11, 12, and 17 (cells under chow diet) were enriched in genes related with apical junction (Figure 4). In contrast, the genes in apical junction were downregulated in the rest clusters which belonged to the condition under LD. Loss of apical junction is responsible for the decrease of epithelial cell-to-cell integrity and could partly contribute to submucosa edema as occurred under LD (van Erpecum et al., 2006). Cluster 13, enriched in genes of *G2m* checkpoint, *E2f* targets, *Myc* targets, were in line with highly proliferation functions. Clusters 4 and 15 were enriched in genes involved in inflammatory response, *Il2*/*stat5* signaling, *Tnfa* singling via *nfkb*. Cluster 3 were enriched in oxidative phosphorylation.

### Changes of Non-epithelial Cells of Gallbladder During the Process of Lithogenesis

Lithogenic diet induced appearance of more non-epithelial cells in comparison with chow diet condition (Figure 3E), suggesting the accompanying submucosal events including micro-vessel genesis, immune cell infiltration, and matrix formation in the gallbladder submucosa wall during the process of lithogenesis.

#### Endothelial Cells

**FIGURE 5:**
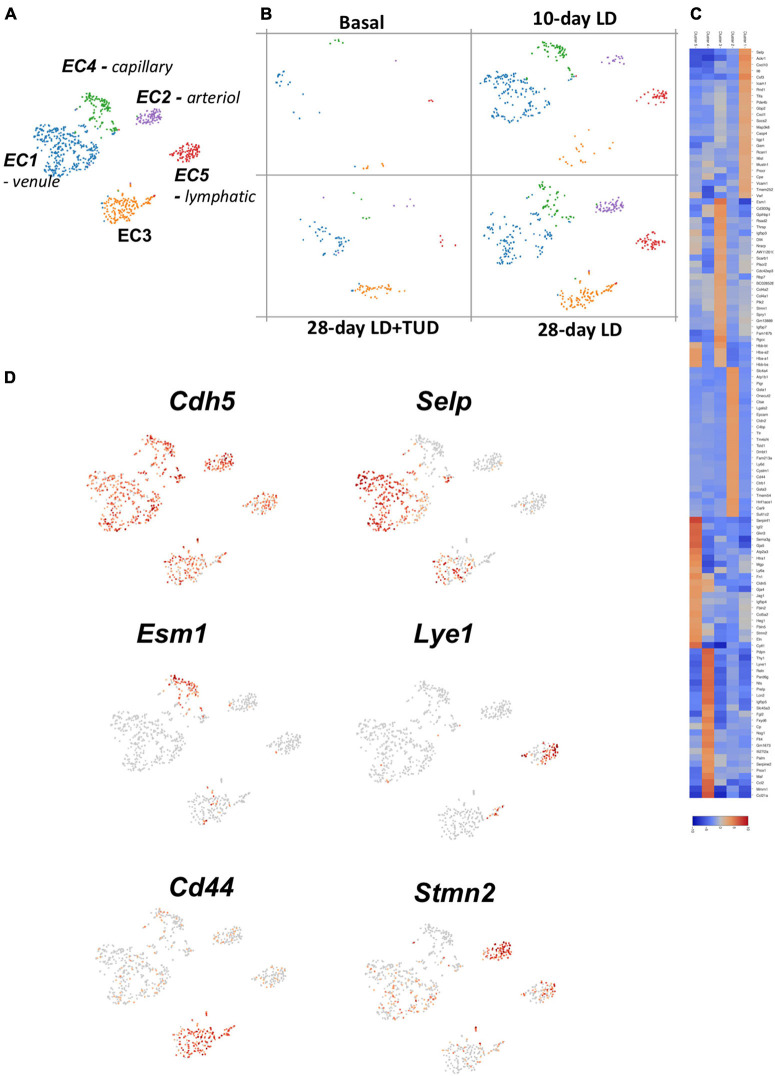
Sub-clustering analysis of the transcriptomes of endothelial cells (ECs) by k-means analysis. **(A)** t-SNE plot identification of subclusters of the EC (cluster 14) which were classified into five types. **(B)** Dynamic changes of ECs during gallstone formation. Cells in all clusters increased rapidly as early as day 10 after LD feeding, especially cluster 1, and constantly increased at day 28 and were mitigated under TUCDA treatment. **(C)** The heatmap indicated enriched expression of specific marker gene expressions of each type of ECs according to their transcriptome pattern. **(D)** t-SNE plot highlighting the sub-clusters of ECs according to representative markers, such as *Cdh5* (pan-marker), *Selp* (venule), *Esm1* (capillary), *Lye1* (lymphatic), *Cd44* (EC3), and *Stmn2* (artierol).

Using K-means analysis, the endothelial cells (cluster 14) could further be sub-clustered into five sub-groups (Figure 5A) reflecting the varieties of their transcriptomes. Cells in all clusters increased rapidly as early as day 10 after LD feeding, especially cluster 1, and constantly increased at day 28 (Figure 5B), indicating presence of active angiogenesis. However, TUDCA feeding inhibited the expanding of endothelial cells. The gene expression heatmap showed differences among groups (Figure 5C) and indicated them to be different types of ECs.

All these ECs were enriched in pan-makers such as *Ptprb* (Nawroth et al., 2002), *Mmrn2* (Christian et al., 2001; Andreuzzi et al., 2017), *Cdh5* (Shah et al., 2016) (Figure 5D and Supplementary Figure 5). Specifically, EC1 were venule ECs with high expression of *Selp* (Blann et al., 2003), *Icam1* and *Vcam1* (Lauridsen et al., 2014). EC2, highly expressed *Stmn2*, and *Eln* (Stoka et al., 2018) belonged to arteriol ECs and EC3 belonged to capillary ECs expressed *Esm1* (Rocha et al., 2014), *Cd300lg* (Takatsu et al., 2006), and *Ramp3* (Yamauchi et al., 2014). EC5 expressed markers of lymphatic EC such as *Lyve1* and *Mmrn1* (Hu et al., 2019). These single-cell transcriptome analyses provide a better resolution of EC heterogeneity and angiogenesis in gallbladder during lithogenesis. EC3 were enriched in genes for cell-cell junction (*Cldn2*, *Cd44*, *Cdh1*) (Rho et al., 2017) and solute transporters (*Slc4a4*). These cells might be responsible for forming the barriers between blood vessels and surrounding tissues controlling the exchange of fluids (Zihni et al., 2016). Importantly, these cells were enriched in *Vegfa* (Supplementary Figure 6), which is a key factor regulating angiogenesis through binding to its receptors-*Vegfr* (Karaman et al., 2018). *Vegfr1, 2, and 3* were differently expressed in EC clusters (Supplementary Figure 6). *Vegr2* was widely expressed in various ECs; in contrast, *Vegfr3* was exclusive expressed in lymphatic ECs and *Vegfr1* was expressed in ECs except lymphatic ECs.

#### Macrophage

**FIGURE 6:**
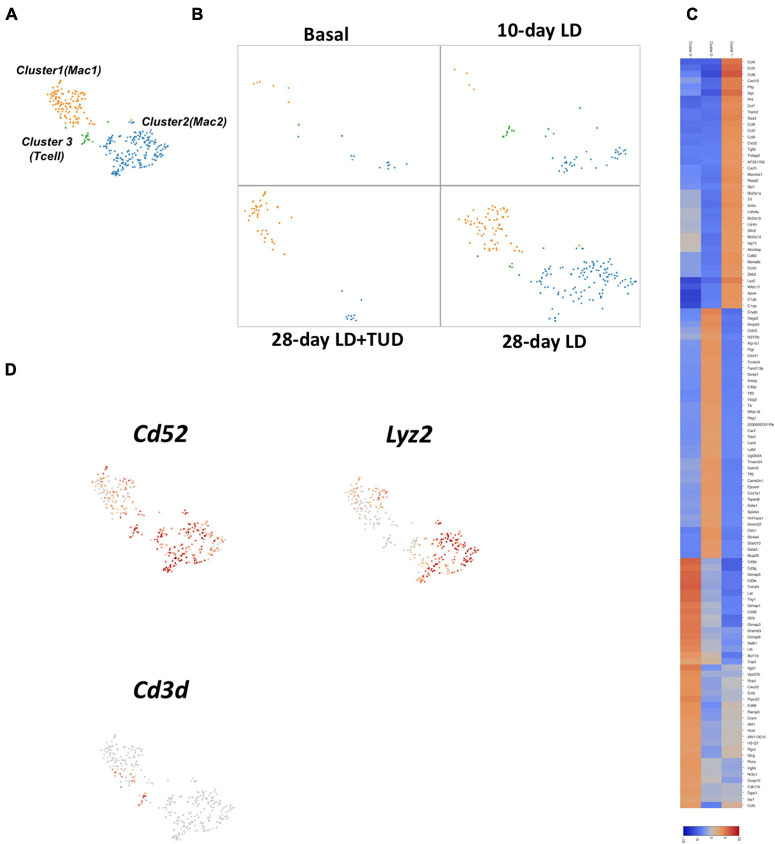
Sub-clustering analysis of the transcriptome pattern of immune cells infiltrated in gallbladder wall. **(A)** t-SNE plot showing three major cell types of immune cells by sub-clustering analysis. **(B)** t-SNE plot showing the dynamic transcriptomic patterns of the immune cells during gallstone formation and TUDCA treatment. **(C)** The heatmap indicated enriched expressions of marker genes in each sub-cluster of immune cells according to their transcriptome pattern. **(D)** t-SNE plot highlighting the sub-clusters of immune cells according to representative markers. Clusters 1 and 2 expressed C52 Campath-1 antigen (*Cd52*). Cluster 3 comprised of a small number of T-cells which expressed markers as *Cd3d* and *Cd3e*.

Immune cells recruitment and infiltration into gallbladder wall was the accompanying event during lithogenesis (Figures 6A,B). The sub-clustering (of cells in cluster 20, Figure 1B) identified at least three types of immune cells according to their transcriptome pattern (Figure 6C). Cluster 3, comprised of a small number of T-cells which expressed markers as *Cd3d* and *Cd3e*, rapidly appeared at day 10 and soon disappeared afterward. Cluster 1 and 2 expressed *C52* Campath-1 antigen (*Cd52*, Figure 6D and Supplementary Figure 7), a glycoprotein of 12 amino acids anchored to glycosylphosphatidylinositol, were widely expressed on the cell surface of immune cells, such as mature lymphocytes, natural killer cells (NK), eosinophils, neutrophils, monocytes/macrophages, and dendritic cells (DCs) (Zhao et al., 2017). LD induced cluster 1 (markers: *Lyz2, C1qb, Cd83*) at day 10 and gradually increased until days 28. This cluster of cells represented macrophages. Usually, macrophages are derived from monocyte infiltrated from the blood stream. In contrast, Cluster 2 cells expressed genes with several aspects of functions. They carry *Ly6d* (a lymphocyte antigen) and *Cd24a*. These cells were active in ceramide and phospholipids metabolism since they were enriched in *Smpd3* (Sphingomyelin phosphodiesterase 3) and *Degs2* [Sphingolipid delta (4)-desaturase/C4-monooxygenase], ion transportation indicated by genes as *Atp1b1* (Sodium/potassium-transporting ATPase subunit beta-1), and *Slc4a4* (Electrogenic sodium bicarbonate cotransporter 1).

In contrast, TUDCA treatment grossly mitigated the infiltration of cluster 1 which were induced by LD, but small amounts of cluster 2 still remained, suggesting their resistance to TUDCA modulation (Figure 6B).

#### Fibroblast Cells

**FIGURE 7:**
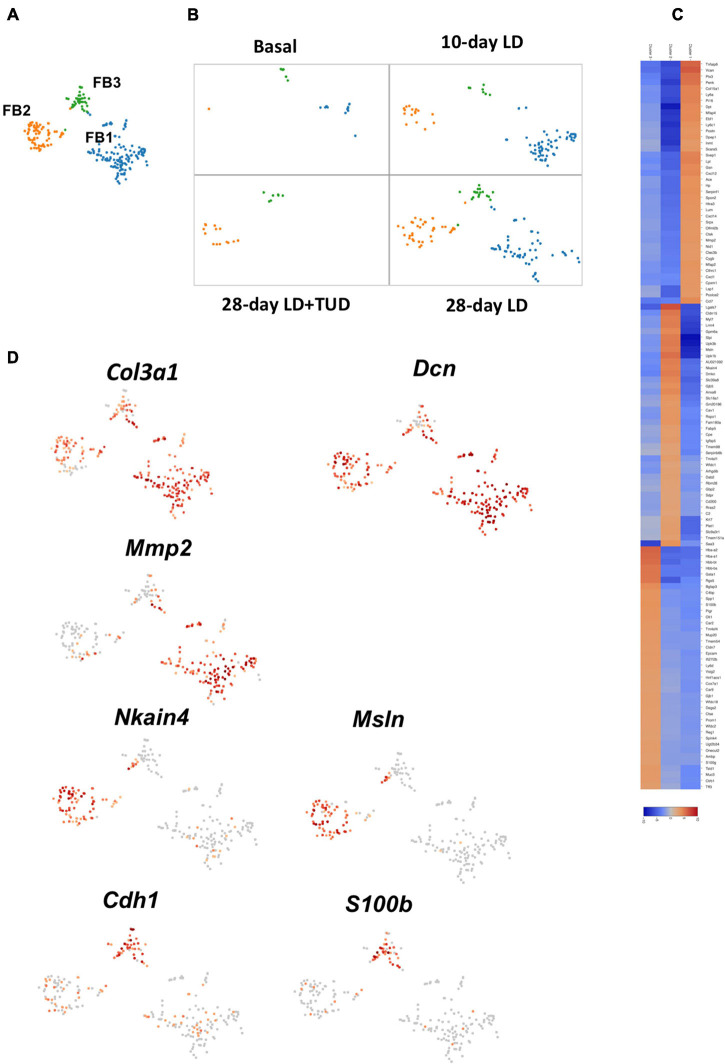
Sub-clustering analysis of the transcriptome pattern of fibroblasts. **(A)** t-SNE plot showing three sub-clusters of fibroblasts in gallbladder. **(B)** t-SNE plot showing the dynamic transcriptomic patterns of fibroblasts during gallstone formation and under TUDCA treatment. They emerged upon LD feeding at day 10 and day 28, but diminished under TUDCA treatment. **(C)** The heatmap indicated enriched expressions of marker genes in each sub-cluster of fibroblasts according to their transcriptome pattern. **(D)** t-SNE plot highlighting the sub-clusters of fibroblasts according to representative markers. Cells in all three sub-clusters expressed pan-markers for fibroblast cell such as collagens (*Col1a1*, *Col3a1*) and proteoglycans (*Dcn*). The expression of calcium binding protein *S100b* suggested the presence of mesenchymal cell to epithelial cell transition.

Fibroblasts (FB) are a group of cells to produce extracellular matrix. Fibroblast cells were limitedly present at basal condition. They emerged early at day 10 upon LD feeding, were persistent until day 28, but receded under TUDCA treatment (Figures 7A,B). K-means analysis sub-clustered them into three clusters with distinctive expression pattern (Figures 7A,C). Cells in all three sub-clusters expressed fibroblast cell specific gene for collagens (*Col1a1*, *Col3a1*) and proteoglycans (*Dcn*) (Figure 7D and Supplementary Figure 8). FB1 was related with ECM producing as they also have high expression of genes such as *Vcan, metal matrix proteinease 2(Mmp2), Col15a2* and glycoproteins (*Dpt*, *Postn*), and fibrillin (*Fbn1*) and lymphocyte antigen Ly6a *(Sca1)*. These cells also expressed high levels of chemokines such as *Cxcl12*, *Ccl7*. FB2 had high level of genes as *Msln*, *Cldn 15* and several transporters as *Slc39a8*, *Slc16a1*, *Nkain4* which were distinctively different than the other FBs. Part of the cells in FB2 also expressed *Myl7*, indicating them to carrying characters of myofibroblasts. In comparison, FB3 carrying epithelium-like characters which had high levels of *Epcam* and *E-cadherin* (*Cdh1*). FB3 cells also carry high level of *Muc1* and *Muc3* expressions which was normally present in epithelium cells of gallbladder. These changes suggesting the present of mesenchymal cell to epithelial cell transition (MET), which might be driven by calcium binding protein S100b (Figure 7D and Supplementary Figure 8), belongs to the family of S100 proteins contain Ca2+-binding EF-hand motifs to interact with calcium signaling involved in cytoskeleton reorganization (Sun et al., 2016; Fritzsche et al., 2017).

#### Cell–Cell Interaction Analysis

**FIGURE 8:**
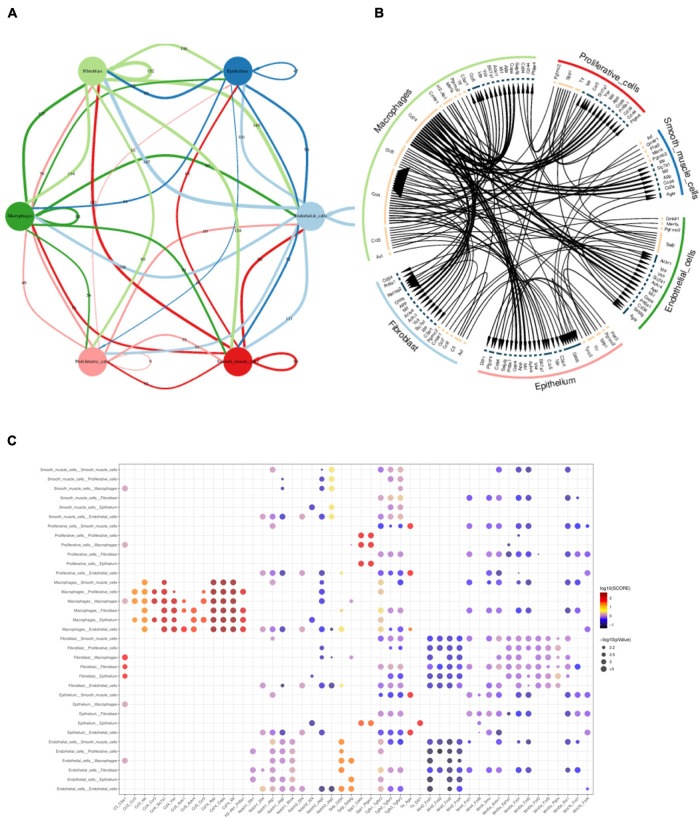
Multi-interactions across and within cell types assessed by CellPhone analysis. **(A)** Summary network showing the level of signaling across and within cell types. Edges were scaled and numbered with the number of ligand-receptor pairs between cell types. **(B)** Chord diagram showing the preferential interactions of ligand-receptors across and within cell types. Edges of lines were scaled according to the interaction scores of ligand-receptor pairs. **(C)** Heatmap showing the interaction scores of ligand-receptor pairs between cell types.

After defining the cell types, we then performed the cell–cell interaction analysis using the literature supported interactions containing ligand–receptor interaction from the chemokine, cytokine, extracellular matrix integrin interactions (Ramilowski et al., 2015) by CellPhone method (Efremova et al., 2020). Multi-ligand-receptor pairs were present between different cell types in gallbladder (Figure 8A and Supplementary Table 2). The most active cell–cell interaction existed between macrophage with epithelial cells, proliferative cells, fibroblast cells, smooth muscle cells, and endothelial cells (Figure 8B). The highest scoring interactions included chemokines such as *Ccl3*, *Ccl4*, *Ccl5* with expressed in macrophage with their ligands *Ccr5* in epithelial cells and proliferative cell (Figure 8C). *Cd74* in macrophages, on the other hand, was highly linked with *Mif (macrophage migration inhibitory factor), App (amyloid b4 protein)* in fibroblast cells, smooth muscle cells, endothelial cells as well as epithelial cells (Figure 8C). In contrast, the canonical signals as *Notch1* or *Wnt* were not strongly linked with their receptors in various cell types.

## Discussion

To our knowledge, this is the first study providing an atlas of RNA transcriptomes of murine gallbladder cells during the process of lithogenesis using sc-RNA sequencing. Our results indicated gallbladder walls were subjected to remodeling during the process of lithogenesis, presenting heterogeneity of gallbladder cells. The major molecular events happened including epithelial damage and repair, and cell proliferation in response to lithogenic bile. Beneath the epithelial wall, immune-cell infiltration, activation of angiogenesis, and extracellular matrix reconstruction were the accompanying events. Various cell types communicated through ligand-receptor pairs, especially macrophages with other cells. Furthermore, we also observed partial reversal effects on gallbladder cell transcriptomes by TUDCA treatment.

The sc-RNA sequencing data in this study provided evidences of changes of cellular transcriptomes in response to lithogenic diet comparing with basal condition. The first distinctive gene signature was decreased expression of genes in apical junction, indicating an impairment of cell to cell junction during lithogenesis which could result in increased permeability of water from bile into gallbladder wall. This was in line with the observation of early edema of gallbladder submucosa after lithogenic diet (van Erpecum et al., 2006). Changes of several aquaporin proteins in the epithelial cells of gallbladder were previously proposed to be responsible for these pathological defects. A possible involvement of aquaporins in the development of gallstones was provided by Paumgartner and Sauerbruch (1991). They reported about the expression of AQP8 in the gallbladder mucosa of humans with small pigment stones without gallbladder inflammation and with similar bile composition as in healthy humans (Paumgartner and Sauerbruch, 1991). This patho-mechanism was further supported by the observation of augmented water absorption from the gallbladder during the initial stages of cholesterol stone formation (Conter et al., 1986; Lee and Choi, 2004). Our sc-RNA sequencing data suggested that the epithelial cells with distinct functions for water absorption was not specified in certain cell clusters since all the proposed aquaporin genes were omni-expressed in gallbladder epithelial cells as we showed.

The second distinctive changes of gallbladder epithelial cells were enrichment of genes in pathways of wound healing in conditions under lithogenic diet. Proliferation of epithelial cells, which appeared well before the occurrence of gallstones (Scott, 1978), together with previous reports of enhanced mucous secretion during lithogenesis (Scott, 1976), made it clear that the injury of gallbladder preceded gallstone formation. The gallbladder reacted to injury at the time before gallstones were forming or formed (Scott, 1978). Several signaling pathways involved in wound healing which are important in regulating these processes, including sonic hedgehog (Zhang et al., 2020), Rho GTPases (Toyokawa et al., 2019), STAT3 (Wang et al., 2020), and Wnt (Burgy and Konigshoff, 2018), were induced.

Third, a number of actively proliferative cells were induced following lithogenic diet. The epithelial cells at basal condition were more at rest state and less active in proliferation. In contrast, proliferation of gallbladder epithelium cells was activated soon after lithogenic diet, much earlier than the occurrence of apparent typical gallstone, providing the hint that this phenomenon was induced by chemical changes in biliary lipids or chemical signals in the epithelium rather than the mechanical stimulation after gallstones formed. Previous studies using isotope labeled precursor of DNA observed increased DNA synthesis in gallbladder epithelium in models such as rabbit fed with dihydrocholesterol-fed (Kaye et al., 1966), guinea pigs under lincomycin treatment (Scott, 1976), or bile duct ligated mice (Scott, 1974). These results indirectly indicated cell proliferation of gallbladder epithelium to be an early and accompanying event during the process of lithogenesis. The early proliferative changes in the gallbladder could occur as early as 2 days after lithogenic diet (Scott, 1978; Putz et al., 1981) with significant increase of total DNA extracted from the entire gallbladder after 2 weeks, whereas visible hypertrophy of the epithelium on the histological sections occurred until 4 weeks after diet induction.

Angiogenesis underneath the epithelial wall of gallbladder was another event happening during lithogenesis. In this study, we were able to identify various ECs as arteriole, venule, capillary ECs, as well as a fraction of lymphatic ECs using their corresponding markers. In one way, angiogenesis was accompanied by the demand for nutrient and oxygen supply in the need of tissue repair and proliferation (DiPietro, 2016). On the other hand, the blood flow provided circulating immunocytes into gallbladder wall as well (Maurer et al., 2009). The EC3 cluster (capillary) were more enriched in *Dll4* expression indicating the involvement of *Dll4-Notch* pathway in the angiogenesis through regulating endothelial cell proliferation, migration, and angiogenic sprouting (Pitulescu et al., 2017). EC2 were enriched in *Vegfra* which could secreted and binding to the *Vegfr2* (*Kdr*) expressed in the rest of the ECs and promoted their proliferation, differentiation, and sprouting (Pitulescu et al., 2017).

Fibroblasts were also involved in the remodeling of the gallbladder wall. They represented a family of related cell types with specialized functions in the synthesis and maintenance of extracellular matrix (Driskell et al., 2013; Rinkevich et al., 2015). They were responsible for modulating ECM through extracellular matrix formation and turnover. In general, they exhibited various degrees of activation including a mix of markers involved in ECM development (*Col1a1*, *Col5a1*), cell–matrix interaction (*Spp1*, *Dcn*), and cell signaling (*Serpine1*, *Postn*). Sub-cluster of FBs (FB2) expressed proteins of muscle contraction (*Acta2*, *Tagln*, *Tpm2*), indicating that they affect the neighboring smooth muscle contraction through paracrine action and disturb gallbladder motor function. This sub-cluster also had the functions for ion transportation like epithelial cells (Skou and Esmann, 1979). Furthermore, a fraction of cells (FB3) had considerable levels of mucin gene expression which was a function of epithelial cells. These cells also had higher level calcium binding protein S100b, a driven protein for mesenchymal cell to epithelial cell transition (Chen et al., 2015). This suggested that MET of mesenchymal cells might occur during the process of lithogenesis.

When we looked upon the lipid absorption of epithelial cells in our sc-RNA sequencing data, we found that the well-known transporters for cholesterol were not specified in certain clusters of epithelial cells. Furthermore, no distinct dynamic expression was observed under lithogenic diet. The sc-RNA sequencing data showed low levels of the expression of these transporter genes, especially those well-known apical membrane transporters such as *Npc1l1*, *Abcg5*, and *Abcg8*, which gave the hint that gallbladder epithelial cells *per se* might not be active in cholesterol absorption from bile or excretion into bile, limiting their capacity to regulate cholesterol contents in bile during lithogenesis. Such phenomenon was not consistent with the results from *in vitro* experiments which showed significant appearance of these transporters under cholesterol induction in cell culture medium. Lithogenic diet could induce accumulation of cholesterol contents in gallbladder wall (Pemsingh et al., 1988). Considering the sc-RNA sequencing data on the known cholesterol transporters, we might deduce that the cholesterol in gallbladder wall might more be originated from peripheral lipoproteins since lithogenic diet markedly elevated plasma cholesterol levels rather than directly absorbed from bile. In the past, more studies focused on the absorption of cholesterol by gallbladder epithelium. For example, the scavenger receptor class B type I (SR-BI) (Miquel et al., 2003) was a potential apical cholesterol transporter expressed in the human gallbladder; megalin, in combination with cubilin, mediated endocytosis of numerous ligands, including HDL/apolipoprotein A-I (apoA-1), were involved in cholesterol absorption by the gallbladder epithelium (Tsaroucha et al., 2008). What we found, here, was that these classical molecules were not actively expressed in epithelial cells, limiting their capacity to regulate biliary cholesterol contents.

At last, we observed partial reversal effect of TUDCA on the overall transcriptomic changes of gallbladder cells. The TUDCA could protect epithelial cells from damages, benefiting epithelial restitution (Goossens and Bailly, 2019). Our current study suggested that promotion of epithelial restitution might also be involved and this hypothesis was supported by an *in vivo* study in mouse model where topical application of TUDCA increased re-epithelialization and the rate and extent of wound closure (Mroz et al., 2018).

## Conclusion

Our results presented heterogeneity of gallbladder cells during the process of lithogenesis and identified major events in epithelial and non-epithelial cells, including proliferation of epithelial cells, infiltration of immune cells, cells involved in angiogenesis, and extracellular matrix modulation. This study also created a reference transcriptome map for murine gallbladder cells through the process of cholesterol gallstone formation, for better understanding how the gallbladder changed dynamically in the pathogenesis of gallstone formation.

## Data Availability Statement

The raw data generated in the manuscript can be found here https://www.ncbi.nlm.nih.gov/geo/query/acc.cgi?acc=GSE179524.

## Ethics Statement

The animal study was reviewed and approved by the Ethical Committee at Shanghai East Hospital.

## Author Contributions

ZJ and AG designed and supervised the study and revised the manuscript. JL, WS, and QLi conducted the study and analyzed the data. QLu provided the technical support. JL, WS, and QLi wrote and revised the draft. All the authors read and approved the final manuscript.

## Conflict of Interest

The authors declare that the research was conducted in the absence of any commercial or financial relationships that could be construed as a potential conflict of interest.

## Publisher’s Note

All claims expressed in this article are solely those of the authors and do not necessarily represent those of their affiliated organizations, or those of the publisher, the editors and the reviewers. Any product that may be evaluated in this article, or claim that may be made by its manufacturer, is not guaranteed or endorsed by the publisher.

## Footnotes

1
https://support.10xgenomics.com/single-cell-gene-expression/software/pipelines/latest/what-is-cell-ranger

2
https://support.10xgenomics.com/single-cell-gene-expression/software/visualization/latest/what-is-loupe-cell-browser

3
https://support.10xgenomics.com/single-cell-gene-expression/software/pipelines/latest/algorithms/overview

4
http://satijalab.org/seurat/

5
https://www.ncbi.nlm.nih.gov/geo/query/acc.cgi?acc=GSE179524

## Supplementary Material

Supplementary Figure 1 Counts and percentage of cells for scRNA-Seq.

Supplementary Figure 2 Heatmap show of markers in each cell cluster of gallbladder cells by scRNA-seq analysis.

Supplementary Figure 3 t-SNE plot highlighting the sub-clusters of epithelial cells according to representative markers. The epithelial cells were enriched in makers for mucin secretion (mucin, *Muc3*, *Muc4*) and water channel (aquaporin 1, *Aqp1*).

Supplementary Figure 4 Violin plots showing the expression level of representative markers across different cluster of cells. **(A)** Proliferative cells (Cluster 13), *Cdca3*; **(B)** Endothelial cells (Cluster 14), *Pecam1*; **(C)** Macrophages (Cluster 20), *C1qa*; **(D)** Fibroblast (Cluster 21), *Dcn*; **(E)** Smooth muscle cells (Cluster 19), *Acta2*; **(F)** Cluster 22, *Rgs13*.

Supplementary Figure 5 Violin plots showing the expression level of representative markers across the different sub-clusters of endothelial cells.

Supplementary Figure 6 t-SNE plot highlighting the sub-clusters of endothelial according to representative markers. *Vegfr1*, *2*, and *3* were differently expressed in EC clusters.

Supplementary Figure 7 Violin plots showing the expression level of representative markers across the different sub-clusters of immune cells.

Supplementary Figure 8 Violin plots showing the expression level of representative markers across the different sub-clusters of fibroblasts.

## Abbreviations

scRNA-seq: single-cell RNA sequencing
LD: lithogenic diet
TUDCA: tauroursodeoxychlic acid
GEM: gel beads in emulsion
t-SNE: t-distributed stochastic neighbor embedding
ECs: endothelial cells
FBs: fibroblast cells
GO: Gene Ontology
GSVA: Gene Set Variant Analysis
NK: natural killer cells
DCs: dendritic cells.
