# Supplementary figures and images for: Single Cell RNA-Sequencing Reveals a Murine Gallbladder Cell Transcriptome Atlas During the Process of Cholesterol Gallstone Formation
Source: Front Cell Dev Biol. 2021 Sep 28;9:714271. doi: 10.3389/fcell.2021.714271 (PMC8505819; doi:10.3389/fcell.2021.714271)

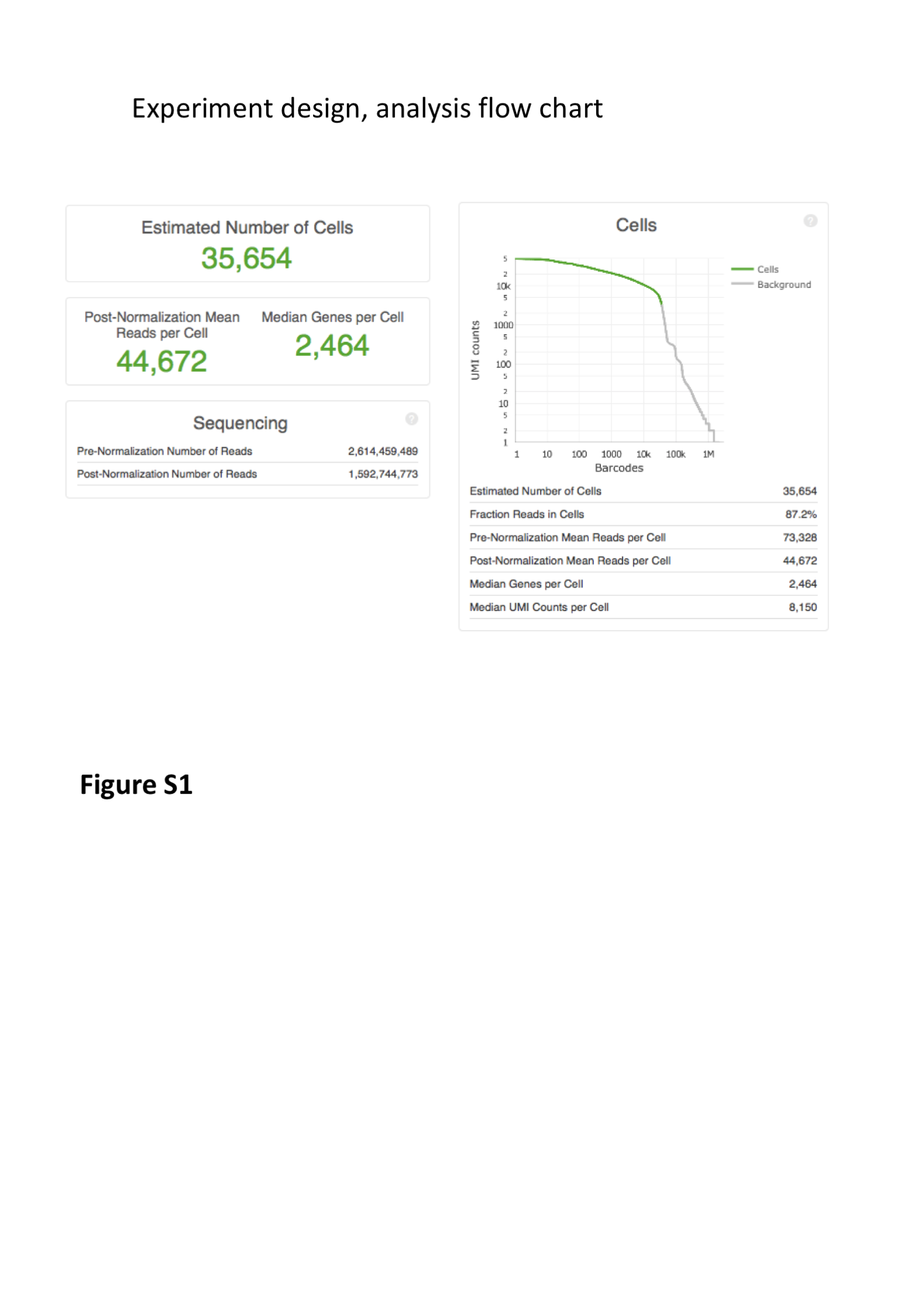

Supplement: Supplementary Figure 1 — Counts and percentage of cells for scRNA-Seq. [file Image_1.TIFF]

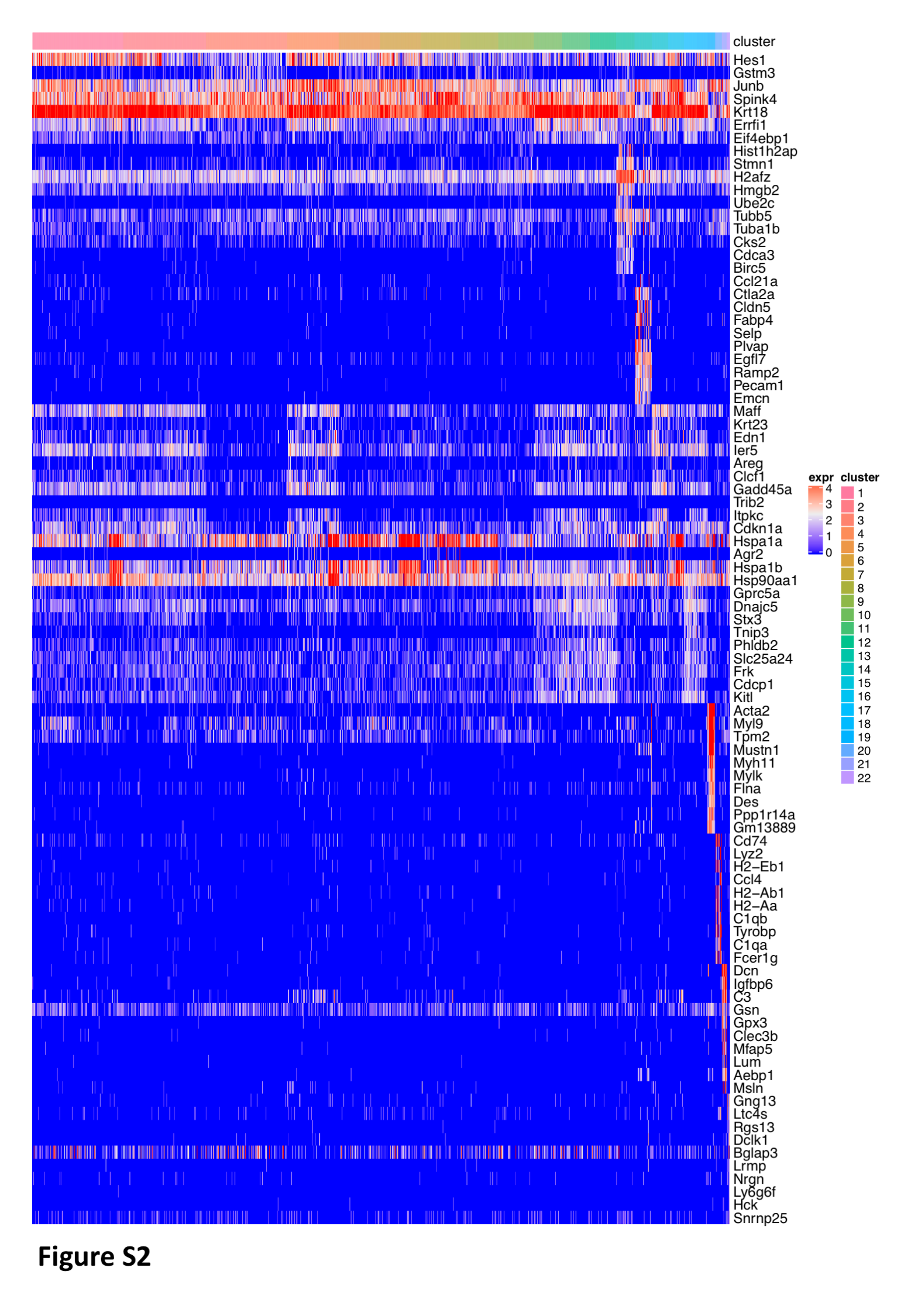

Supplement: Supplementary Figure 2 — Heatmap show of markers in each cell cluster of gallbladder cells by scRNA-seq analysis. [file Image_2.TIFF]

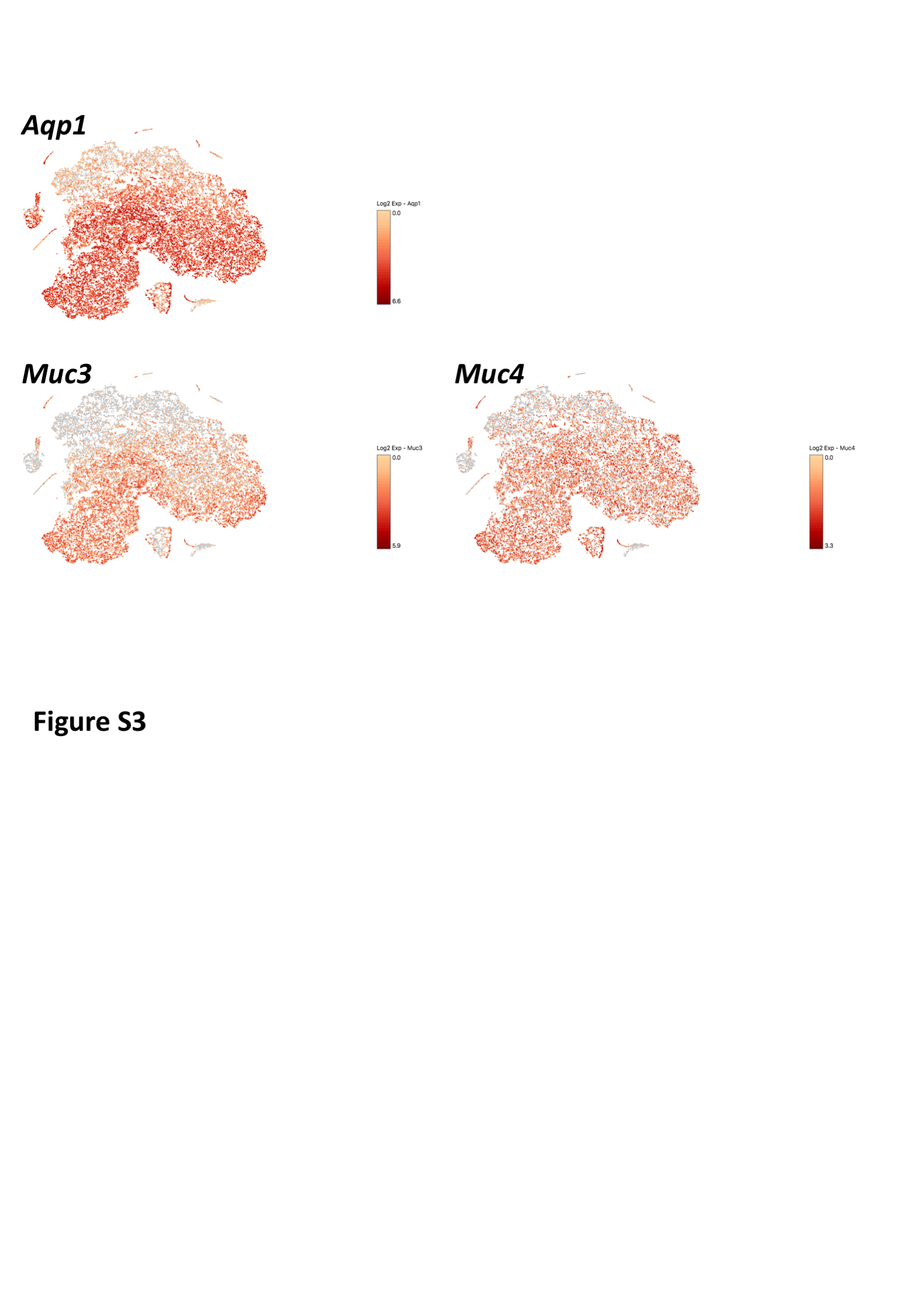

Supplement: Supplementary Figure 3 — t-SNE plot highlighting the sub-clusters of epithelial cells according to representative markers. The epithelial cells were enriched in makers for mucin secretion (mucin, Muc3, Muc4) and water channel (aquaporin 1, Aqp1). [file Image_3.TIFF]

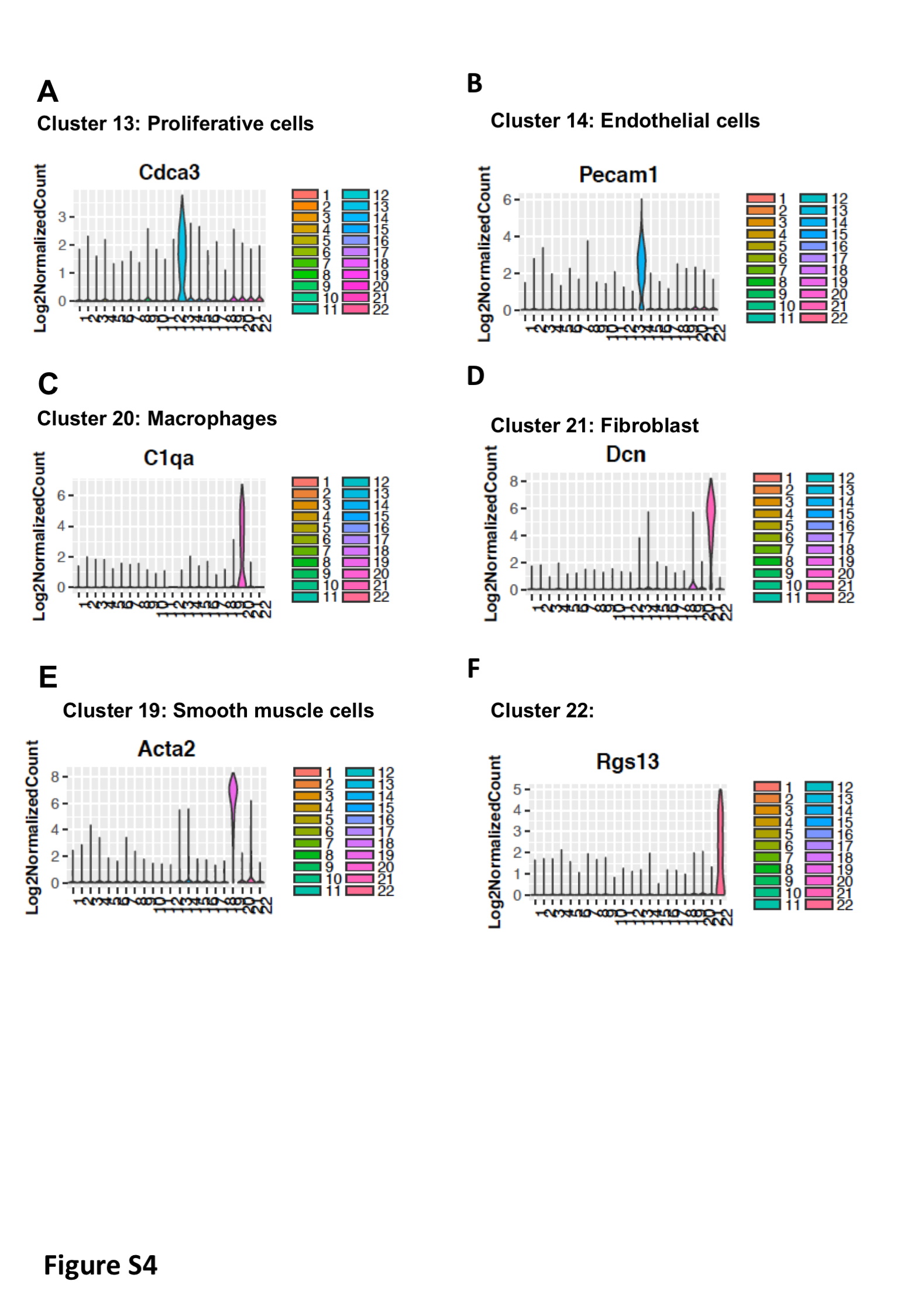

Supplement: Supplementary Figure 4 — Violin plots showing the expression level of representative markers across different cluster of cells. (A) Proliferative cells (Cluster 13), Cdca3; (B) Endothelial cells (Cluster 14), Pecam1; (C) Macrophages (Cluster 20), C1qa; (D) Fibroblast (Cluster 21), Dcn; (E) Smooth muscle cells (Cluster 19), Acta2; (F) Cluster 22, Rgs13. [file Image_4.TIFF]

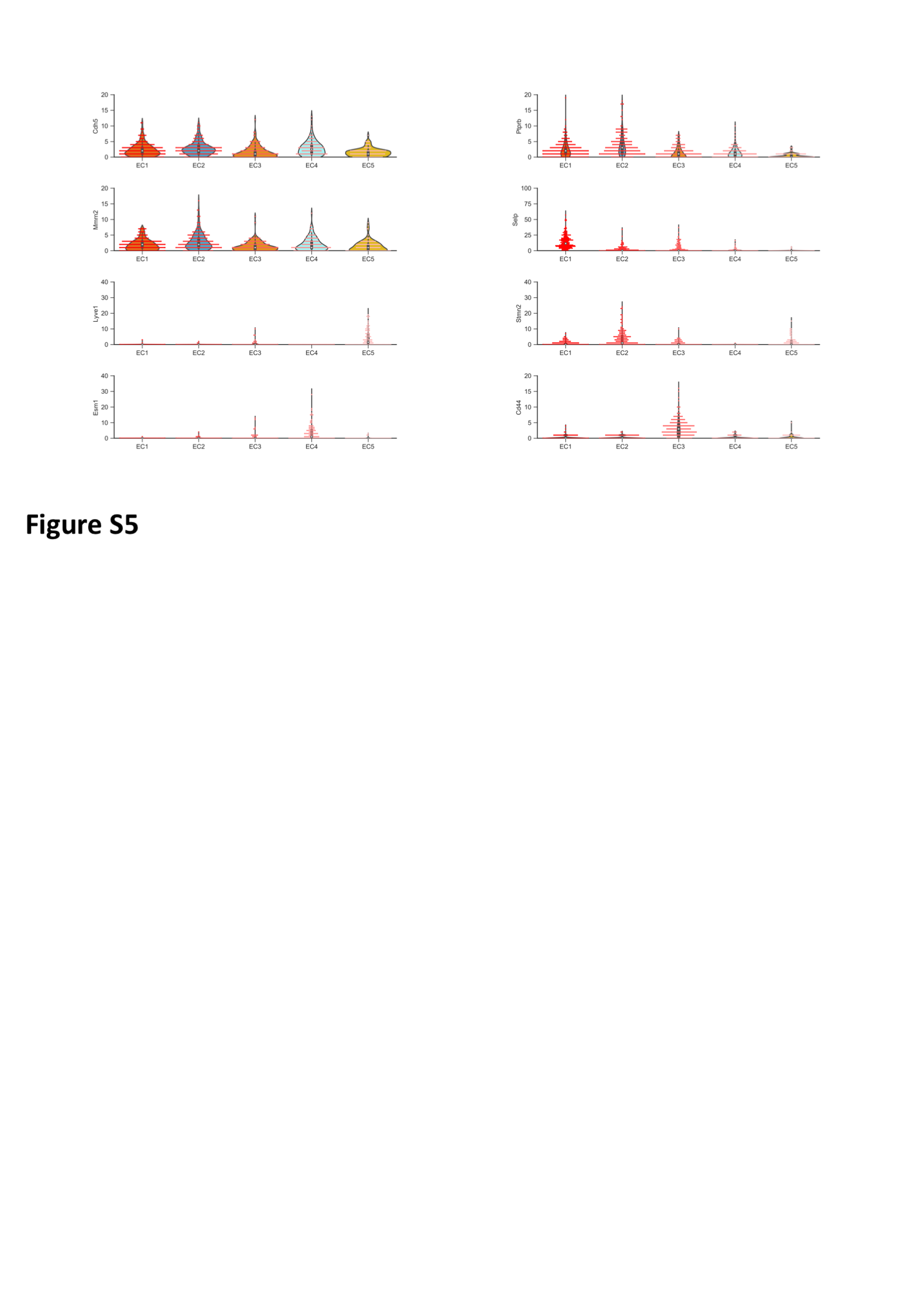

Supplement: Supplementary Figure 5 — Violin plots showing the expression level of representative markers across the different sub-clusters of endothelial cells. [file Image_5.TIFF]

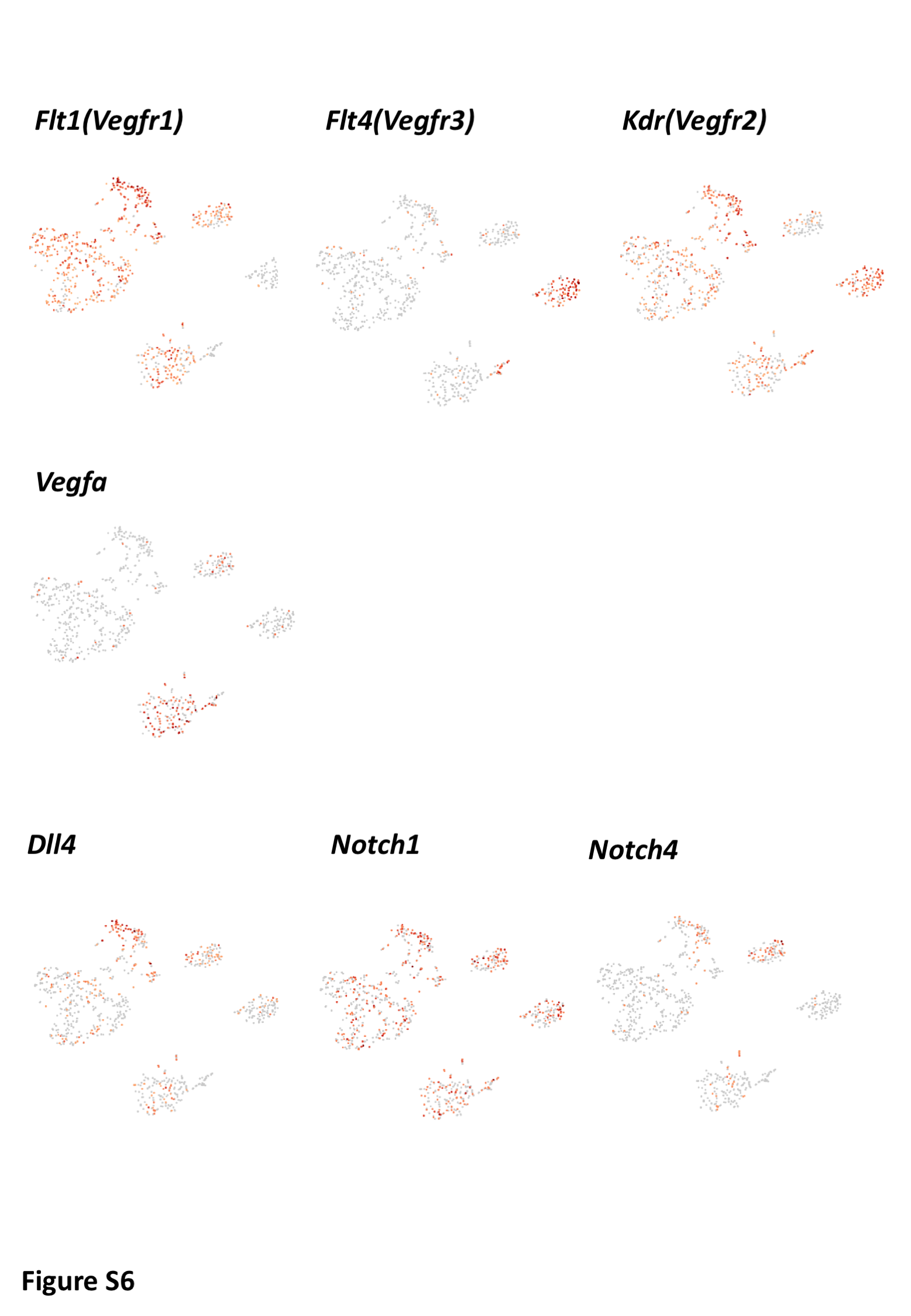

Supplement: Supplementary Figure 6 — t-SNE plot highlighting the sub-clusters of endothelial according to representative markers. Vegfr1, 2, and 3 were differently expressed in EC clusters. [file Image_6.TIFF]

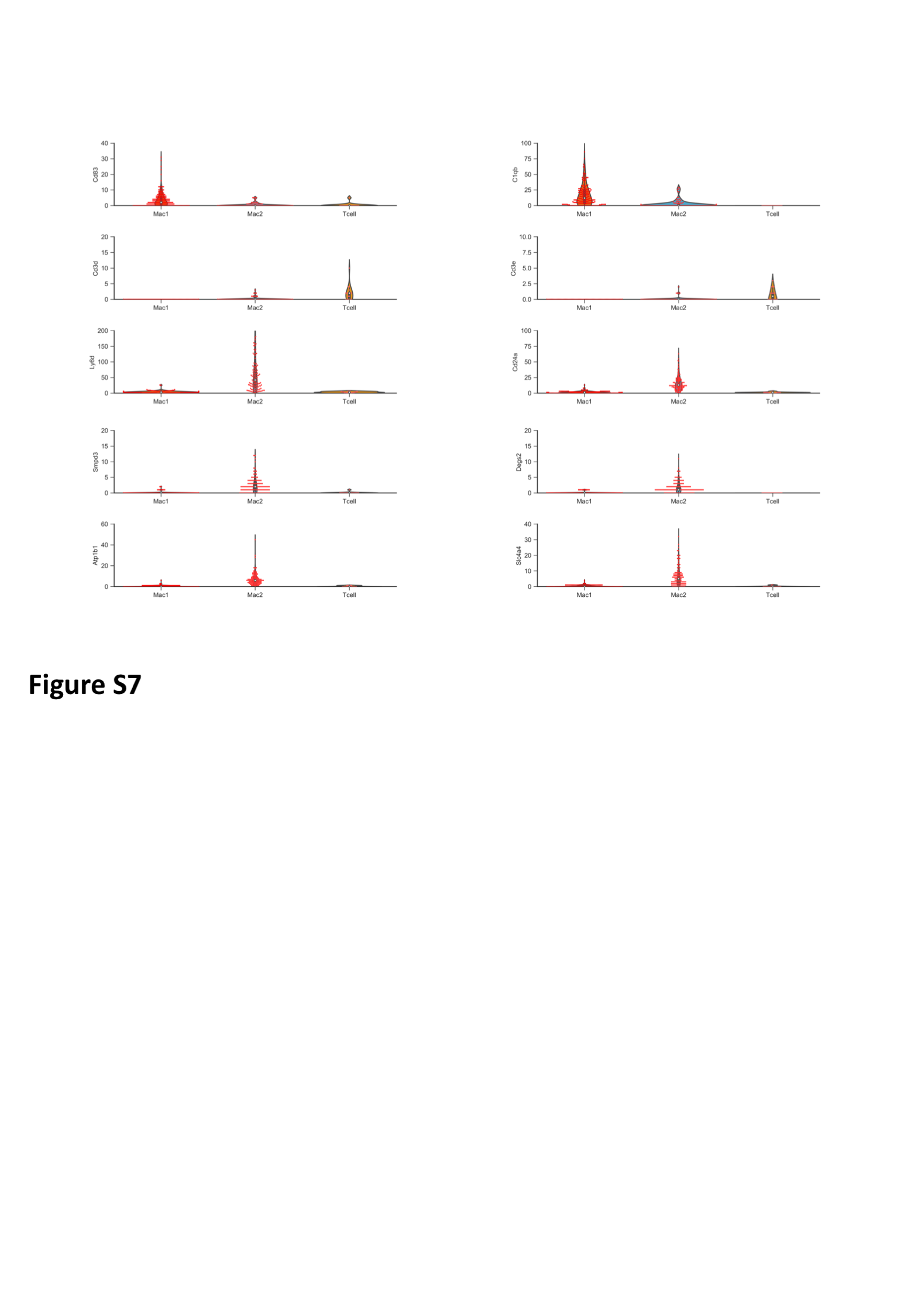

Supplement: Supplementary Figure 7 — Violin plots showing the expression level of representative markers across the different sub-clusters of immune cells. [file Image_7.TIFF]

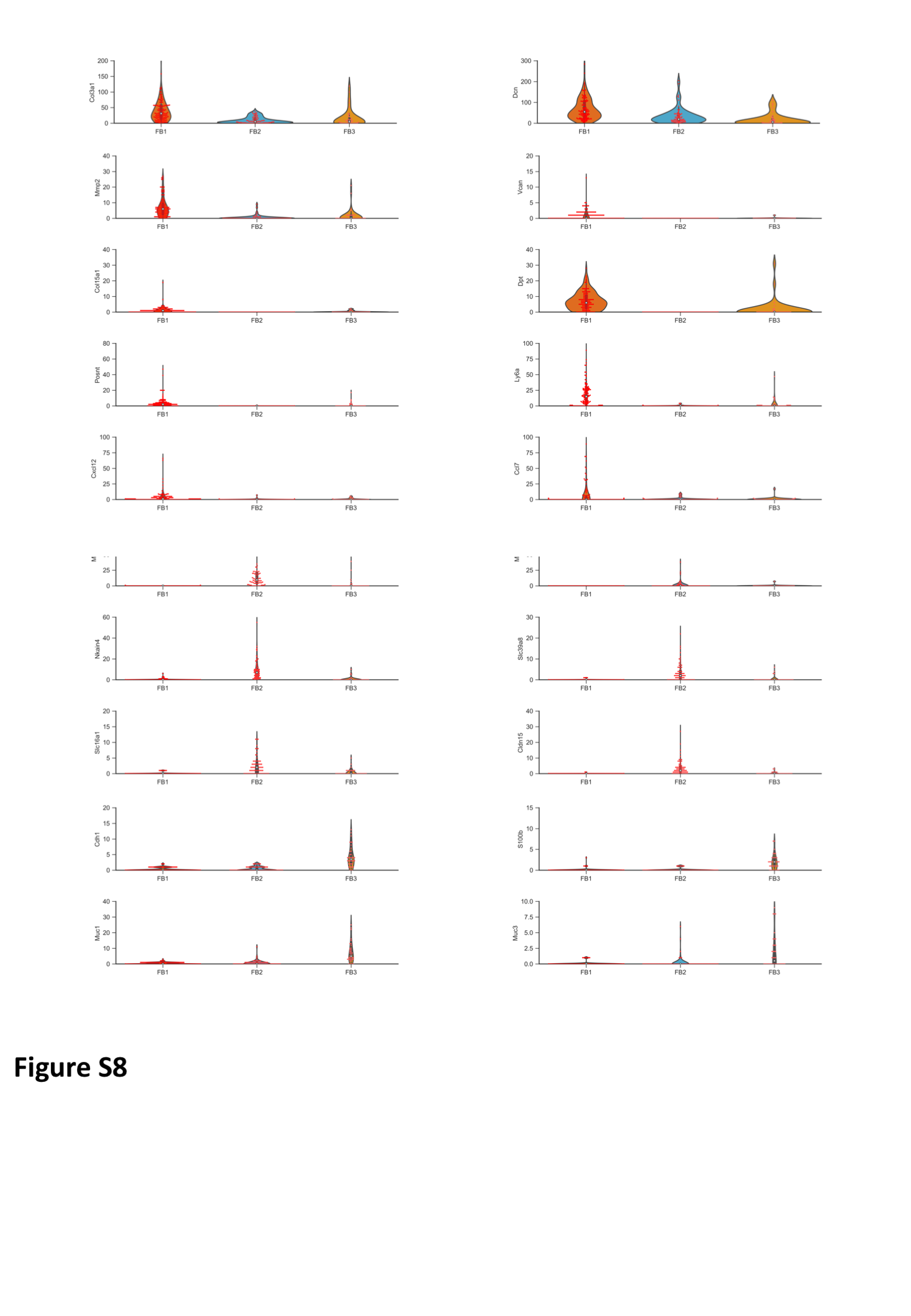

Supplement: Supplementary Figure 8 — Violin plots showing the expression level of representative markers across the different sub-clusters of fibroblasts. [file Image_8.TIFF]
